# Treatment of Unruptured, Saccular, Anterior Choroidal Artery Aneurysms with Flow Diversion: A Single Centre Experience

**DOI:** 10.1007/s00062-018-0677-1

**Published:** 2018-03-07

**Authors:** P. Bhogal, O. Ganslandt, H. Bäzner, H. Henkes, M. Aguilar Perez

**Affiliations:** 1 Neuroradiological Clinic, Neurocenter, Klinikum Stuttgart, Kriegsbergstraße 60, 70174 Stuttgart, Germany; 2 Neurosurgical Clinic, Neurocenter, Klinikum Stuttgart, Stuttgart, Germany; 3 Neurological Clinic, Neurocenter, Klinikum Stuttgart, Stuttgart, Germany; 4 Medical Faculty, University Duisburg-Essen, Essen, Germany

**Keywords:** Aneurysm, Flow diverter

## Abstract

**Background:**

The region of the brain supplied by the anterior choroidal artery (AChoA) is exquisitely eloquent. Aneurysms arising at or close to the origin of the vessel are not uncommon and damage or occlusion to the vessel can result in devastating consequences. The optimal treatment strategy is yet to be determined.

**Objective:**

We sought to determine the efficacy of flow diversion for the treatment of unruptured AChoA aneurysms.

**Method:**

A retrospective review of our prospectively maintained database was performed to identify all patients with unruptured aneurysms of the AChoA between March 2009 and May 2017. The fundus size, number and type of flow-diverting stent (FD), complications and follow-up data were recorded.

**Results:**

We identified 30 patients (60% female), average age 52.8 ± 10.8 years (range 27–73), with 30 aneurysms. The aneurysms were generally small with a mean fundus diameter of 3.4 mm (range 1–7 mm). Early angiographic follow-up data were available for all patients at which point 15 aneurysms were completely occluded (50%). Delayed angiographic follow-up was available in 24 patients and occlusion was seen in 21 patients (87.5%). Of the patients one developed transient ischemic symptoms after interruption of the antiplatelet medication and another patient had a small embolic infarct with transient symptoms in the periprocedural period.

**Conclusion:**

Flow diversion can be used to successfully treat aneurysms of the AChoA. The treatment carries a high rate of technical and radiological success with a good safety profile.

## Introduction

The anterior choroidal artery (AChoA) supplies an extensive and eloquent territory, which includes the posterior two-thirds of the internal capsule, the adjacent optic radiations, medial portion of the globus pallidus, caudate tail, uncus, hippocampal head, amygdala, piriform cortex and part of the lateral geniculate nucleus [1–5]. Occlusion of the AChoA can therefore result in severe neurological deficits for patients with the development of hemiparesis, hemi-hypesthesia, and hemi-anopsia with motor and sensory deficits being most common [2, 6–10].

Aneurysms of the AChoA account for 4% of all intracranial aneurysms [11]. As with aneurysms elsewhere, both surgical clipping and endovascular treatment options are available. Surgical clipping of AChoA aneurysms carries a high mortality and morbidity with rates varying between 5% and 50% in the literature and principally due to ischemic stroke [12–18]. In 2004 Piotin et al. [11] published a series of 18 patients with AChoA aneurysms treated endovascularly with coils (*n* = 12), balloon remodeling technique (*n* = 4) or stent-assisted coiling (*n* = 1). In this series, there was one treatment-related death secondary to aneurysm perforation and one transient contralateral hemiparesis that resolved within 24 h. This paper demonstrated that standard endovascular techniques were amenable to the treatment of AChoA aneurysms with a good safety profile.

More recently, the introduction of flow diverters (FD) has increased the armamentarium of interventional neuroradiologists. Naturally, there was initial trepidation regarding the coverage of small vessels and their potential occlusion, especially important vessels such as the AChoA, despite animal experimental data to the contrary. Neki et al. [19] recently published their retrospective analysis of 20 patients in whom the AChoA was unavoidably covered during the endovascular management of intracranial aneurysms. They showed that no patients complained of transient or permanent clinical symptoms related to AChoA occlusion and that the vessel remained patent without any flow changes.

With the goal of assessing the efficacy and complication rate of flow diversion treatment for unruptured AChoA aneurysms we retrospectively studied 32 consecutive patients treated endovascularly with either the Pipeline embolization device (PED, Medtronic, Minneapolis, MN, USA) or the p64 flow remodeling device (phenox, Bochum, Germany).

## Methods

### Patient Population

We searched our prospectively maintained database, for all patients treated in our institution with unruptured, saccular AChoA aneurysms between March 2009 and May 2017. For each patient we recorded demographic data, clinical presentation, location of the aneurysm, therapeutic intervention, immediate angiographic and clinical result, and clinical and radiological follow-up information. The data was entered into the prospectively collected computer database for each institution.

### Classification of AChoA Aneurysms

The analysis of the aneurysm characteristics was performed by the senior neurointerventionalist (HH). The AChoA aneurysms were lesions that could be seen arising directly from the AChoA or adjacent to the AchoA at the branching point. The size of the aneurysms was recorded as the greatest sac diameter. Aneurysms were carefully assessed and aneurysms of the posterior communicating artery or terminal internal carotid artery (ICA) were excluded.

### Endovascular Treatment

All treatments were performed with the patient under general anesthesia. The two commercially available FDs, PED (Medtronic, Irvine, CA, USA) and p64 (phenox, Bochum, Germany) were used. All the aneurysms were treated with only a single type of FD, either a PED or a p64 (there were no cases where both types of FD were used in the same patient) although in four cases the aneurysms had been previously unsuccessfully treated with coils and one with the Medina embolic device (Medtronic). In two patients coils were used during the same procedure. Patient informed consent was obtained before the procedure in all cases. The selection of FD was dependent on availability and the operators’ judgement. Selection of the FD was initially based on availability. Initially only the PED was available, however, after the p64 gained the CE approval it was also available for use in our department. Furthermore, in our experience, the p64 offers advantages in that it can be completely deployed and re-sheathed to allow repositioning alongside improved visibility compared to the PED [20].

All patients received dual antiplatelet therapy with either 100 mg acetylsalicylic acid (ASA) daily and 75 mg clopidogrel for at least 3 days or a loading dose of 500 mg ASA and 600 mg clopidogrel on the day prior to the treatment. The effectiveness of the antiplatelet regime was tested using the Multiplate analyzer (Roche, Town, Country or state abbreviation for USA) and/or VerifyNow (Accriva, San Diego, CA, USA). The post-procedural antiplatelet regime consisted of 75 mg clopidogrel per day continued for 12 months following treatment and 100 mg ASA per day continued for life. Patients resilient to clopidogrel received either 10 mg prasugrel or 2 × 90 mg ticagrelor per day.

All procedures were performed via the femoral route using a 6 Fr access system as standard. All procedures were performed under heparin anticoagulation with a 5000 IU bolus dose at the start of the procedure and subsequent 1000 IU bolus doses every hour to maintain the activated clotting time between 2–2.5 times the baseline.

### Procedural Assessment and Follow-Up

Patency and flow characteristics within the ICA and AChoA were assessed angiographically immediately after placement of the FD and during follow-up. In-stent stenosis with neointimal hyperplasia was graded as none (0%), mild (<50%), moderate (50–75%) and severe (>75%; [21]). Aneurysms were graded using the 3‑point Raymond-Roy classification (RRC; [22]). Grading was performed by a diagnostic neuroradiologist within the department that was not involved with the procedure itself.

Neurological examinations were performed to evaluate for potential ischemic or hemorrhagic complications in the postoperative period (<24 h post-procedure) and at each subsequent follow-up. The neurological assessment was performed by a member of the neurosurgical team and a member of the interventional neuroradiology team. The modified Rankin Score (mRS) was recorded pre-operatively, immediately post-operatively and at all subsequent follow-ups.

## Results

We identified 30 patients that met our inclusion criteria. Of the 30 patients 18 were female (60%) with an average age of 52.8 ± 10.8 years (range, 27–73 years) at presentation. A total of 30 saccular aneurysms were treated, 16 (53%) of which were on the left side. In four cases the aneurysm had been previously unsuccessfully treated with coils. The aneurysms were generally small with a mean average maximum fundus diameter of 3.4 mm (range 1–7 mm).

The PEDs were used to treat 4 of the aneurysms and the p64 was used to treat the remaining 26 aneurysms. There were no cases where both a PED and a p64 were used. In 3 patients treated with the PED more than 1 stent was used (3 telescoped PED in 2 patients and 2 telescoped PED in 1 patient). In 5 patients treated with the p64, 2 telescoped stents were placed one of which was due to inappropriate positioning of the first p64. In the remainder of the cases only a single p64 was placed.

**Fig. 1:**
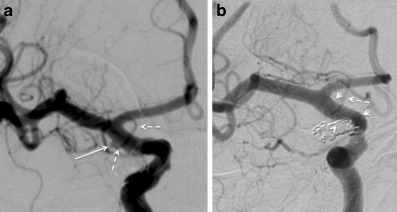
A patient with a right-sided AChoA aneurysm treated with a single p64. The preoperative angiography (**a**) shows the small 2 mm aneurysm (*white arrow*) with the AChoA clearly visible (*dashed white arrows*). The postoperative follow-up angiogram (**b**) performed 3 months later shows complete occlusion of the aneurysm (*white arrow head*) and a patent AChoA (*dashed white arrow*). The markers for the p64 can also be seen (*short white arrows*); *AchoA* anterior choroidal artery

**Fig. 2:**
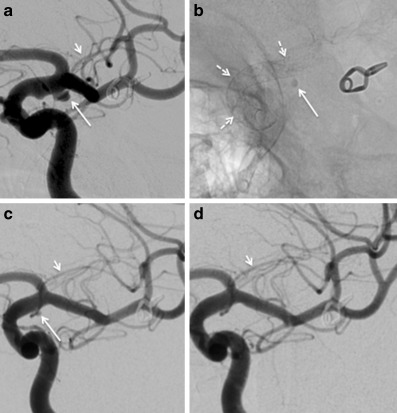
A patient treated with a single PED for a 3 mm AChoA aneurysm. The preoperative angiogram (**a**) shows the aneurysm (*long white arrow*) with the AChoA clearly seen (*short white arrow*). The unsubtracted image (**b**) following deployment of the PED (*dashed white arrows*) shows contrast stagnation within the aneurysm (*long white arrow*). Follow-up angiography at 3 months shows a small remnant (**c**) with complete occlusion of the aneurysm at delayed angiography performed after 15 months (**d**); *AchoA* anterior choroidal artery, *PED* Pipeline Embolisation Device

Early angiographic follow-up data were obtained for all aneurysms at a mean average of 3.1 months after the procedure. At initial follow-up 15 of the aneurysms showed complete occlusion (RRC I) and 8 aneurysms showed a small neck remnant (RRC II; Fig. 1). The remaining aneurysms showed continued contrast opacification (RRC III). At mid-term follow-up at a mean of 9.9 months after treatment, was available for 24 patients, 19 aneurysms showed complete occlusion (RRC I) and 2 showed a small remnant (RRC II; Fig. 2). The remaining 6 patients did not attend for follow-up angiography. At delayed follow-up available in 14 patients and performed at mean average of 31.9 months, complete occlusion was seen in all the aneurysms. In total 23/30 aneurysms were occluded on follow-up angiography (76.6%). Of the 24 patients with delayed angiography 21 aneurysms were occluded (87.5%). The AchoA was patent in all cases. The results are summarized in Table 1.

**Table 1:** Patient demographics, aneurysms characteristics and follow-up data

			Aneurysm Characteristic										
Patient	Age (years)	Sex	Laterality	Fundus max. dimension	Neck width	Previous treatment	No. FDs	First F/U (months)	Last F/U (months)	Occluded	AChoA Patent	mRS Pre-op.	mRS Post-op.
1	73	f	l	5	3	0	1 p64	2 months	9 months	Y	Y	2	2
2	43	f	l	2	2	0	1 p64	3 months	20 months	Y	Y	0	0
3	53	m	l	4	3	Coils	1 p64	3 months	25 months	Y	Y	0	0
4	40	f	l	6	6	Coils	1 p64	3 months	9 months	Y	Y	2	2
5	53	f	r	2	2	0	1 p64	3 months	21 months	Y	Y	0	0
6	60	f	l	3	3	0	1 PED	3 months	15 months	Y	Y	0	0
7	56	f	r	4	2	0	2 PED	4 months	54 months	Y	Y	0	0
8	46	m	l	3	2	0	1 p64	3 months	12 months	Y	Y	1	1
9	68	f	l	3	3	0	1 p64	4 months	10 months	*N*	Y	1	1
10	58	f	r	5	3	0	1 p64	3 months	10 months	Y	Y	0	0
11	45	m	l	3	3	0	3 PED	3 months	32 months	Y	Y	0	0
12	61	f	r	5	5	0	1 p64	4 months	NA	*N*	Y	0	0
13	57	m	r	4	3	0	1 p64	3 months	10 months	Y	Y	0	0
14	41	f	l	4	3	0	1 p64	3 months	8 months	Y	Y	0	0
15	64	m	l	3	2	0	1 p64	3 months	10 months	*N*	Y	0	0
16	64	m	r	4	2	0	1 p64	3 months	NA	*N*	Y	0	0
17	27	f	r	3	4	0	1 p64	3 months	21 months	Y	Y	0	0
18	53	m	l	2	2	0	2 p64	3 months	12 months	Y	Y	2	2
19	47	f	r	2	3	0	1 p64	3 months	NA	Y	Y	0	0
20	61	f	l	2	3	0	2 p64	6 months	11 months	*N*	Y	0	0
21	52	m	r	2	1.5	0	1 p64	3 months	10 months	Y	Y	0	0
22	30	f	r	2.5	2.5	Coils	2 p64	3 months	9 months	Y	Y	0	0
23	39	m	l	1	1	0	2 p64	3 months	11 months	Y	Y	0	1
24	53	f	l	3	3	0	3 PED	4 months	47 months	Y	Y	0	0
25	62	m	r	7	6	Coils	1 p64	3 months	NA	Y	Y	2	2
26	64	f	r	7	5	0	1 p64	6 days	NA	*N*	Y	0	0
27	61	m	r	2	2	0	1 p64	5 months	NA	*N*	Y	0	0
28	47	f	l	2	2	0	2 p64	2 months	7 months	Y	Y	0	0
29	59	f	l	2	2	0	1 p64	2 months	6 months	Y	Y	1	0
30	42	m	r	4	2	0	1 p64	4 months	10 months	Y	Y	2	2

*m* male, *f* female, *y* yes, *n* no, *NA* not applicable, *F/U* follow-up, *mRS* modified Rankin Score

Nine patients demonstrated intimal hyperplasia on initial follow-up angiography (8 patients <50%, 1 patient 50–75%, all asymptomatic). In 5 cases the intimal hyperplasia resolved and in 1 patient the intimal hyperplasia improved during the follow-up period, in 2 it remained stable (<50%) and 1 follow-up is pending.

### Complications

In one patient, there was a small embolic ischemic lesion that represented the only case of periprocedural morbidity. A further patient developed transient ischemic embolic symptoms during the follow-up period after a transient interruption of the antiplatelet medication. Both patients returned to baseline neurology. There were no cases of vessel rupture or dissection. There were no cases of delayed rupture or morbidity.

## Discussion

The AChoA artery supplies an extremely eloquent area of the brain with several potential anastomotic pathways known to exist with the posterior choroidal arteries, the interpeduncular plexus and the posterior communicating arteries [4, 23]. These anastomotic connections are not easily assessed and therefore their presence or ability to undertake supply of the AChoA territory, if this vessel is impaired, is difficult to judge. For these reasons the most feared consequence of treatment of aneurysms arising from AChoA is infarction within the territory of this vessel that can but does not always, result in a devastating outcome for patients [24, 25]. Friedman et al. [17] reported a case series of 51 AChoA aneurysms in 50 patients, 33 of whom presented with acute subarachnoid hemorrhage (SAH), 3 patients died, 2 from treatment-related complications and 8 patients (16%) demonstrated clinical and computed tomography (CT) evidence of infarction within the AChoA territory. More recently Li et al. [13] presented their single centre experience of 102 patients with AChoA aneurysms treated with microneurosurgical clipping. In total 106 aneurysms were treated with an overall surgical mortality of 7% and major surgical morbidity of 12%. A similar rate of AChoA infarction (15%) to that by Friedman et al. [17] was reported. To our knowledge, Bohnstedt et al. [26] have published the largest surgical series in the English literature. Of the 127 aneurysms, treated 112 underwent microsurgical clipping, 5 aneurysmal wrapping and 2 surgical exploration. The remaining 8 aneurysms were treated endovascularly with coils. The postoperative ischemic complication rate in those patients that underwent microneurosurgical treatment was 12.6% (15 of 119 patients), 4 of whom died during hospitalization or shortly thereafter. Interestingly, just over half of the patients (8 of 15) that demonstrated AChoA territory infarction showed persistent flow within the artery. This group also reported that the use of temporary clipping more often led to postoperative ischemia and that the average number of times the ICA was temporarily occluded was statistically significant in leading to postoperative ischemia, 1.51 for non-ischemia, 2.56 for ischemia (*p* = 0.007). Clip repositioning was also statistically significantly associated with postoperative ischemia. This goes to show that even temporary interruptions to the flow with the AChoA can have disastrous effects likely due to the inability of the collateral supply to supply the metabolic demands of the territory; however, we acknowledge that a direct comparison with our results or the results from other studies using flow diversion, could be erroneous and a well-designed randomized controlled trial (RCT) would be the only way to provide an accurate answer on the merits of either technique.

The endovascular management of AChoA aneurysms has until recently consisted principally of coiling. In 2004, Piotin et al. [11] published their series of 18 consecutive patients that underwent endovascular treatment of AChoA berry aneurysms. In this series 14 patients presented with SAH and two of the aneurysms were associated with arteriovenous malformations (AVM). There was one death secondary to rupture of the aneurysm during the coiling procedure with another patient developing a transient contralateral hemiparesis. More recently, Gimonet et al. [27] described a three-catheter technique to treat AChoA with coils. This involved placing one catheter in the aneurysm, one inside the AChoA itself to protect it and finally a balloon catheter in the ICA. This technique was used to treat 6 aneurysms, all of which were small (mean fundus size 2 × 2 × 2 mm), and in all patients the aneurysms were successfully occluded with no ischemic complications or aneurysm rupture. Other endovascular coiling techniques have also been described [28] as have single center series looking at the risk of complications from the treatment of these aneurysms [29].

Kim et al. [30] sought to compare the outcomes of patients undergoing either surgical or endovascular treatment of AChoA aneurysms. In their retrospective study 38 patients were treated with coiling and 35 with clipping. Of the patients in the clipping cohort, four developed permanent contralateral hemiparesis due to AChoA infarction with a further patient developing a 3rd cranial nerve palsy. In the coiling cohort two patients developed temporary contralateral hemiparesis that resolved completely. The authors suggest that the overall outcome and prevention of repeat hemorrhage for both groups was similar but with a significantly lower incidence of AChoA territory infarction within those patients treated endovascularly.

The advent of FD has provided a new treatment option for these aneurysms and although there was initial concern that covering the AChoA may result in infarction this does not seem to be the case. In the series by Neki et al. [19] there were no documented cases of AChoA territory infarction and in all the cases (85%) with follow-up angiography the AChoA remained patent. This is in agreement with our study and whilst the AChoA is a small artery it is still larger than the pore sizes of FD [4, 31, 32]. At nominal diameter the PED pore size is between 0.02–0.05 mm^2^ [31] with the area of the origin of the AChoA varying from 0.4 to 1.1 mm^2^ (mean 0.9 mm^2^; [4]). Therefore, even though the stent struts are likely to cross the origin of the AChoA, as long as adequate flow is maintained in the parent artery and the AChoA occlusion is unlikely to occur. The alteration in flow within the aneurysms that allows occlusion to occur with the simultaneous preservation of flow within the AChoA, as shown in our study, highlights the potential for this treatment option for these otherwise difficult to treat and high-risk aneurysms. Other groups have also recently published their results of FD involving the anterior choroidal artery. Raz et al. [33] analyzed 157 patients who had at least a PED covering the origin of the AChoA with 29 patients meeting this criteria. At angiographic follow-up (mean 15.1 months) 28 AChoA were patent with anterograde flow. In a single case there was occlusion of the artery and retrograde reconstitution via the medial lenticulostriate and posterolateral choroidal arteries. Interestingly the arteries in this case arose from the fundus of the aneurysm and 3 PEDs were used for the treatment of the aneurysm and although the patient had very transient symptoms consistent with a AChoA syndrome, these symptoms lasted for 5 min only. Similarly Brinjikji et al. [34] presented the findings of 15 consecutive patients where a PED was placed covering the ostium of the AChoA. In this series none of the aneurysms treated actually arose from the AChoA and 12 of the aneurysms were unruptured. In this study, immediate postoperative angiography showed anterograde flow in all the covered AChoA. Of the patients 1 died 10 days after treatment secondary to an intraparenchymal hemorrhage but angiography performed 1 day after the procedure showed patency of the AChoA. Of the remaining 14 aneurysms, the AChoA remained patent in 13 and in 1 patient the artery was occluded (mean follow-up 12 months) with no symptoms reported in this patient. Of the patients eight had follow-up magnetic resonance imaging (MRI) or CT imaging and none of the patients demonstrated infarction within the AChoA territory. The study of Rangel-Castilla et al. [35] also reviewed the occlusion of the AChoA as well as other covered branches. In their series of 82 patients, none of the covered AChoA were occluded at follow-up; however, they reported occlusion of other covered branches, such as the ophthalmic artery (10.5%), posterior communicating artery (10.7%, all adult type) and all the covered anterior cerebral artery (ACA) (*n* = 2). Although there were no clinical consequences to the occlusion of any of these branches the authors suggest that the use of multiple FD may contribute to branch occlusion. In our series none of the covered AChoA were occluded at follow-up and there was no evidence of infarction within the territory of the artery.

The main limitations of our study are its retrospective design and the relatively small number of patients. Additionally, as the aneurysms in our case series are all <7 mm, the applicability to larger aneurysms is uncertain. Furthermore, as all the aneurysms are saccular we are unsure of the applicability of the technique to fusiform aneurysms. We also acknowledge that a direct comparison with our results and the results from other studies using FD with surgical series could be erroneous and a well-designed RCT would be the only way to provide an accurate answer on the merits of either technique to treat these lesions.

## Conclusion

The results of this study suggest that the use of FD for the treatment of unruptured AChoA aneurysms is safe and carries a high rate of technical and radiological success.

## References

[1] Feekes JA, Hsu SW, Chaloupka JC, Cassell MD (2005). Tertiary microvascular territories define lacunar infarcts in the basal ganglia. Ann Neurol.

[2] Ghika JA, Bogousslavsky J, Regli F (1990). Deep perforators from the carotid system. Template of the vascular territories. Arch Neurol.

[3] Hamoir XL, Grandin CB, Peeters A, Robert A, Cosnard G, Duprez T (2004). MRI of hyperacute stroke in the AChA territory. Eur Radiol.

[4] Rhoton AL, Fujii K, Fradd B (1979). Microsurgical anatomy of the anterior choroidal artery. Surg Neurol.

[5] Rosner SS, Rhoton AL, Ono M, Barry M (1984). Microsurgical anatomy of the anterior perforating arteries. J Neurosurg..

[6] Sohn H, Kang D-W, Kwon SU, Kim JS (2013). Anterior choroidal artery territory infarction: lesions confined to versus beyond the internal capsule. Cerebrovasc Dis.

[7] Hupperts RM, Lodder J, Heuts-van Raak EP, Kessels F (1994). Infarcts in the anterior choroidal artery territory. Anatomical distribution, clinical syndromes, presumed pathogenesis and early outcome. Brain.

[8] Bruno A, Graff-Radford NR, Biller J, Adams HP (1989). Anterior choroidal artery territory infarction: a small vessel disease. Stroke.

[9] Helgason C, Caplan LR, Goodwin J, Hedges T (1986). Anterior choroidal artery-territory infarction. Report of cases and review. Arch Neurol..

[10] Fisher M, Lingley JF, Blumenfeld A, Felice K (1989). Anterior choroidal artery territory infarction and small-vessel disease. Stroke.

[11] Piotin M, Mounayer C, Spelle L, Williams MT, Moret J (2004). Endovascular treatment of anterior choroidal artery aneurysms. AJNR Am. J. Neuroradiol..

[12] Drake CG, Vanderlinden RG, Amacher AL (1968). Carotid-choroidal aneurysms. J Neurosurg.

[13] Li J, Mukherjee R, Lan Z, Liu Y, He M (2012). Microneurosurgical management of anterior choroidal artery aneurysms: a 16-year institutional experience of 102 patients. Neurol Res.

[14] Yasargil MG, Yonas H, Gasser JC (1978). Anterior choroidal artery aneurysms: their anatomy and surgical significance. Surg Neurol.

[15] Viale GL, Pau A (1979). Carotid-choroidal aneurysms: remarks on surgical treatment and outcome. Surg Neurol.

[16] Shibata Y, Fujita S, Kawaguchi T, Hosoda K, Komatsu H, Tamaki N (2000). Use of microvascular Doppler sonography in aneurysm surgery on the anterior choroidal artery. Neurol. Med. Chir. (Tokyo).

[17] Friedman JA, Pichelmann MA, Piepgras DG, Atkinson JL, Maher CO, Meyer FB, Hansen KK (2001). Ischemic complications of surgery for anterior choroidal artery aneurysms. J Neurosurg..

[18] Suzuki K, Kodama N, Sasaki T, Matsumoto M, Konno Y, Sakuma J, Oinuma M, Murakawa M (2003). Intraoperative monitoring of blood flow insufficiency in the anterior choroidal artery during aneurysm surgery. J Neurosurg..

[19] Neki H, Caroff J, Jittapiromsak P, Benachour N, Mihalea C, Ikka L, Moret J, Spelle L (2015). Patency of the anterior choroidal artery covered with a flow-diverter stent. J Neurosurg..

[20] Bhogal P, Hellstern V, Bäzner H, Ganslandt O, Henkes H, Aguilar Pérez M (2017). The use of flow diverting stents to treat para-ophthalmic aneurysms. Front Neurol.

[21] Aguilar Pérez M, Bhogal P, Henkes E, Ganslandt O, Bäzner H, Henkes H. In-stent stenosis after p64 flow diverter treatment. Clin Neuroradiol. 2017 May 9. [Epub ahead of print] 10.1007/s00062-017-0591-y PMC6245240 28488025

[22] Roy D, Milot G, Raymond J (2001). Endovascular treatment of unruptured aneurysms. Stroke.

[23] Carpenter MB, Noback CR, Moss ML (1954). The anterior choroidal artery; its origins course, distribution, and variations. AMA Arch. Neurol. Psychiatry.

[24] Das K, Benzil DL, Rovit RL, Murali R, Couldwell WT (1998). Irving S. Cooper (1922–1985): a pioneer in functional neurosurgery. J Neurosurg.

[25] Perria L, Viale GL, Rivano C (1971). Further remarks on the surgical treatment of carotid-choroidal aneurysms. Acta Neurochir. (Wien).

[26] Bohnstedt BN, Kemp WJ, Li Y, Payner TD, Horner TG, Leipzig TJ, Cohen-Gadol AA (2013). Surgical treatment of 127 anterior choroidal artery aneurysms: a cohort study of resultant ischemic complications. Neurosurgery.

[27] Gimonet H, Desal H-A, Mosimann PJ, Stracke P, Daumas-Duport B, Lintia-Gaultier A, Bourcier R, Chapot R (2016). A new endovascular technique for small anterior choroidal artery aneurysms. A consecutive series using the 3‑catheter-protective technique. J Neuroradiol.

[28] Heo YJ, Yang KH, Jung SC, Park JC, Lee DH (2016). “Two-coil technique” for embolization of small internal carotid artery aneurysms incorporating the origin of the anterior choroidal artery. Interv Neuroradiol.

[29] André A, Boch AL, Di Maria F, Nouet A, Sourour N, Clémenceau S, Gabrieli J, Degos V, Zeghal C, Chiras J, Cornu P, Clarençon F. Complication risk factors in anterior choroidal artery aneurysm treatment. Clin Neuroradiol. 2017 Mar 20. [Epub ahead of print] 10.1007/s00062-017-0575-y 28321460

[30] Kim BM, Kim DI, Shin YS, Chung EC, Kim DJ, Suh SH, Kim SY, Park SI, Choi CS, Won YS (2008). Clinical outcome and ischemic complication after treatment of anterior choroidal artery aneurysm: comparison between surgical clipping and endovascular coiling. AJNR Am J Neuroradiol.

[31] Fischer S, Vajda Z, Aguilar PM, Schmid E, Hopf N, Bäzner H, Henkes H (2012). Pipeline embolization device (PED) for neurovascular reconstruction: initial experience in the treatment of 101 intracranial aneurysms and dissections. Neuroradiology.

[32] Kulcsár Z, Ernemann U, Wetzel SG, Bock A, Goericke S, Panagiotopoulos V, Forsting M, Ruefenacht DA, Wanke I (2010). High-profile flow diverter (silk) implantation in the basilar artery: efficacy in the treatment of aneurysms and the role of the perforators. Stroke.

[33] Raz E, Shapiro M, Becske T, Zumofen DW, Tanweer O, Potts MB, Riina HA, Nelson PK (2015). Anterior choroidal artery patency and clinical follow-up after coverage with the pipeline embolization device. AJNR Am J Neuroradiol.

[34] Brinjikji W, Kallmes DF, Cloft HJ, Lanzino G (2015). Patency of the anterior choroidal artery after flow-diversion treatment of internal carotid artery aneurysms. AJNR Am. J. Neuroradiol..

[35] Rangel-Castilla L, Munich SA, Jaleel N, Cress MC, Krishna C, Sonig A, Snyder KV, Siddiqui AH, Levy EI (2016). Patency of anterior circulation branch vessels after Pipeline embolization: longer-term results from 82 aneurysm cases. J Neurosurg..

